# Marketing strategies of the female-only gym industry: A case-based industry perspective

**DOI:** 10.3389/fpsyg.2022.928882

**Published:** 2022-09-23

**Authors:** Fong-Jia Wang, Chia-Huei Hsiao, Tao-Tien Hsiung

**Affiliations:** ^1^Office of Physical Education, Tamkang University, New Taipei, Taiwan; ^2^Department of Leisure and Sport Management, National Taipei University, Taipei, Taiwan; ^3^Department of Turkish Language and Culture, National Chengchi University, Taipei, Taiwan

**Keywords:** case study, innovation services, women’s health, gender, fitness services

## Abstract

Female customers are an important market for fitness centers. This study aims to examine successful fitness training health models used by female fitness clubs. A case study approach and the interview method were used to collect data (i.e., face-to-face interviews) regarding the marketing strategies of female fitness clubs. Purposive sampling was employed to select executives working at the headquarters of a female-only gym, and semi-structured interviews were conducted to provide a context for fitness behaviors and to discuss the business’ marketing mix. Six managers at Curves (four female and two male) across six different positions were interviewed. This study found that Curves’ most significant difference compared to other private fitness clubs is that it makes women feel comfortable. Through charitable activities combined with promotions, women feel as if the gym empathizes with them. This study is unique in that it is one of the first studies to explore the business model of the female fitness club. In particular, this research suggests that sports organizations should focus their services strategies on female exercise demands. The findings of this study will support the development of marketing strategies to promote the growth of women’s health as a priority within the fitness industry.

## Introduction

Due to increased awareness of health and wellness, fitness centers have become more prominent in the sports market sector (Wang and Chiu, 2022). In addition to strengthening the body and enhancing health, exercise can sculpt the body and improve one’s self-image (Farrell et al., 2011; Shaw, 2012; Andreasson and Johansson, 2013). In addition, through sports and leisure activities, one can expand interpersonal relationships and build a personal network. Participating in sports and leisure activities even becomes a promoter of social class mobility, thereby creating and accumulating social capital. The increasing amount of people partaking in sports and fitness activities has brought social and economic benefits, making it a thriving business (Chang, 2020; Andreasson and Johansson, 2021).

Fueled by social media, sports and leisure activities have become an integral part of daily life in modern society. Different cultural backgrounds, ages, and genders can achieve diversity in different sports and leisure activities (Andreasson and Johansson, 2013). Therefore, sports organizations, clubs, and sports centers launch services or courses to attract different consumers. At the same time, those sports entities strive to provide higher quality services to improve consumer satisfaction (Avourdiadou and Theodorakis, 2014; Teik, 2015; Zhou et al., 2018). Through the application of marketing strategies, we can create different blue oceans in the red ocean of many sports industries.

Historically, the sports field has been dominated by men, as has military service (Armstrong, 1999; Beetles and Harris, 2005). However, women now enjoy higher economic autonomy, more job opportunities, and recognized technical expertise, and their needs differ from those of men. In addition, the physical activity interests and needs of the female population differ from those of the male population (Funk et al., 2003; Avourdiadou and Theodorakis, 2014; Haro-González et al., 2018). A previous study has also mentioned that “sport in the past belonged to men – its future belongs to all human beings!” (Pfister, 2010, p. 245). Therefore, if enterprises do not abandon the ingrained male-oriented marketing mindset of the past, they will not survive in today’s highly competitive market (Farrell et al., 2011; Dodds et al., 2015; Howie and Campbell, 2015).

In addition, due to differences in congenital conditions, women and men also have significant differences in the choice of sports participation and leisure activities (Howie and Campbell, 2015). For example, men may prefer collision-like body exercise, while women may mostly choose milder exercise. However, in the fitness market, women do not need to compete with men. Therefore, the goal of women’s participation in fitness may not be the same as that of men (Biddle et al., 2001). Men may go to the gym to train their muscles, while women may go to the gym to lose weight, remain fit, and attain their desired body shape (Fink, 2015; Gregg and Bedard, 2016; Hutson, 2016; Lee et al., 2018). Furthermore, looks can be seen as a fashion symbol that motivates the modern woman to go to the gym.

The rising power of female consumers, especially the generation of Asian women as the leading economic force, cannot be ignored (Conlin et al., 2014). In recent years, studies have demonstrated the importance of marketing to women and have begun to analyze the consumer behaviors of female consumers. Furthermore, researchers emphasize the particularity of women in the field of sports and leisure activities (Cuneen and Claussen, 1999; Chang, 2020; Andreasson and Johansson, 2021). To improve women’s health, foster sports empowerment, and construct a friendly environment in which women can play sports and leisure activities, the Sports Administration of the Ministry of Education in Taiwan implemented the first and the world’s second white paper on the promotion of women’s sports in 2016 (Hsu et al., 2020). This showed that the Taiwanese government also realized the growing need for sports and leisure activities for the female market.

The gender dimension is one of the variables that is often used as a distinguishing condition in the field of consumer behavior. Women are guided by communal goals to connect with others and cultivate harmonious relationships. Women may be expected to be family-oriented, cooperative, intrinsically positive, and risk-averse. They may also be more likely to enjoy the arts and engage in activities that foster social connection, but these are general tendencies. Gender roles still vary due to individual differences (Papadimitriou et al., 2004). In addition, female consumers pay more attention to the relevance of brands than men do. Women build relationships wherever they go, and products are “communication items” for women. That is to say, women do not think that a commodity is just a physical object but think that it is a medium that allows people to communicate with objects and between people (Roussel et al., 2003; Xiong et al., 2020). The purpose of women’s communication is to build relationships and initiate interaction and emotional exchange. Therefore, to develop the female customer market, analyze the characteristics of female consumers, and then grasp the factors that affect female consumption decision-making is quite essential.

According to Andreasson and Johansson (2021), when companies formulate marketing strategies for female customers, they should be aware of the feelings of each individual customer rather than attempting to apply the same approach to all women. In order to stand out in today’s ever-changing and highly competitive market, companies should expel the existing stereotypes, accept new female-oriented trends, and replace old-fashioned marketing strategies with new ideas (e.g., ingrained male-oriented marketing mindset) (Kotler, 2000). The study had already shown that not all women reproduce male behaviors. This circumstance can expose a particular and new female sports culture (Shaw, 2012; Xiong, 2019). In addition, studies have found that females receive more social attention when participating in physical exercise (Tombs and McColl-Kennedy, 2010; Xiong et al., 2020). As a result, several sports and fitness service businesses are considering new marketing strategies that target only women. These businesses are considering spaces, activities, and services that meet the needs of individuals, which has led to the creation of specialized sports services and fitness centers for women (Roussel et al., 2003). The fitness industry is becoming increasingly competitive, requiring service suppliers to come across the altering needs of their consumers (Jane et al., 2018). Servicescape differentiation—directing the needs of segmented markets—enables gyms to attract new markets (Ghobadian et al., 2014). Females who practice fitness sports, in particular, are one such group with particular desires that could progress from this approach. Overall, companies, gyms, or any other enterprises within the fitness industry should explore marketing strategies aimed at women (Hardin et al., 2008).

However, most studies have focused on consumer satisfaction and service quality (Wang and Chiu, 2022). Some discuss women’s requirements for sports and leisure activities. Research on understanding the marketing strategies specifically designed for women, as well as the key success factors of their operations from a business owner’s perspective, is lacking (Pope, 2012; Andreasson and Johansson, 2021). Therefore, this study aims to examine successful fitness training health models used by female fitness clubs in Taiwan. Drawing on the service fitness literature, this study examined the female-only gym’s model of fitness marketing to females. In this way, we intend to show how the power of the innovation market is manifested through marketing strategies directed at females.

## Materials and methods

To gain an in-depth understanding of a fitness club’s business model, marketing strategy, and key factors for business success from the perspective of an operator, two components of data collection were used in this study. The first was document analysis, which included examining Curves’ printed materials and terms and conditions. The second was a series of semi-structured interviews with staff members of a Curves club. Curves were chosen as the subject of this case study because it was the only chain of female-only fitness centers in Taiwan at the time.

### Case of curves

Curves originated in Harrington, Texas, United States, in 1992 (Dobbins et al., 2012; IHRSA, 2018). It was founded by Gary Heavin. The business stemmed from the grief of his mother’s sudden death due to illness. Gary Heavin was determined to protect women’s health, so he chose to study in the Department of Medicine. During his studies, he found that medical treatment alone could not save women like his mother (Garcia-Fernandez et al., 2014). Therefore, he tried to find other ways to protect women and believed that exercise could improve women’s physical condition and avoid sudden death. His idea of founding Curves was “No Men, No Makeup, No Mirrors” (IHRSA, 2018). At Curves, everyone is unique and gathered there for different reasons. It was a female-only fitness center where the female community could relax and encourage each other to exercise with friends. Curves has been investing in women’s weight management worldwide for more than 10 years. As an industry leader, they always strive to make women healthy and happy. From the birth of the first Curves store in 1992, it has gradually developed to this day. Through continuous research and development and improvement, an effective motion system, Curves circular motion, has been created (IHRSA, 2018). Gary Heavin developed a circular exercise training program (Packianathan and Kyungro, 2000; Dobbins et al., 2012; Naredla and Babu, 2018). Twelve hydraulic exercise equipment designed for women can increase the body’s basal metabolic rate. Through the automatic adjustment of the body load through the speed of different users, the female group can perform exercise training according to their own rhythm.

Through simple, time-saving, pleasant, and effective circular movements, Curves has successfully created a set of exercise methods that meet the needs of modern women (Garcia-Fernandez et al., 2014). Curves, a chain of female-only gyms, recognized and harnessed the demands of the female consumer group and created a “blue ocean” for women, making itself a well-known international gym chain. Curves uses a 3 M business strategy (No Men, No Mirrors, and No Makeup) and applies a 3F marketing strategy having fun working out with family and friends (Fun), fast access to the gym (Fast), and effectiveness in improving fitness (Fitness) (Dobbins et al., 2012). Curves has managed to take advantage of women’s workout needs and develop a chain of female-only fitness centers with its 3 M business strategy and 3 F marketing strategy. Female customers love sharing and need companionship, and Curves has taken advantage of the opportunity of word-of-mouth advertising among its female customers. It was able to foster interpersonal influence, which in turn enabled it to establish a stable customer network (Garcia-Fernandez et al., 2014).

Gary Heavin advocated that more female groups can easily and continue to exercise, opened the Curves franchise operation system in 1995, and now has set up Curves branches in more than 80 countries around the world (IHRSA, 2018). At present, the number of global members has exceeded 4 million people. Curves entered Taiwan in 2007. It is the first female-only gym in Taiwan. Currently, there are more than 140 branches in Taiwan with more than 50,000 members (Wang and Chiu, 2022). Curves is a fitness environment tailored for women. All members and coaches are women, which separates the market from the traditional fitness industry so that the female group no longer needs to worry about the free movement of others. Curves creates a pleasant sports atmosphere and meets women’s social needs. Through like-minded sports partners participating in sports training together to inspire each other and make members happy to exercise (Dobbins et al., 2012). On the other hand, based on the above, Curves is undoubtedly a special place, and the ability to obtain sports results in a short time is the reason why Curves is supported and favored by the modern female group. By understanding and mastering women’s needs, successfully building a kingdom of women’s sports and fitness is a key element of Curves’ successful marketing operations.

### Participants

This research aims to explore the business model and marketing strategy formulation used by Curves. Therefore, the participants were set within the company’s high-level managers and executives who design Curves’ marketing strategies. Consumer opinions are not included. There are six managers of Curves in total (four female and two male) holding six different positions (i.e., general manager, deputy general manager, chief marketing manager, chief brand manager, chief human resource manager, and chief finance manager; Table 1) who were interviewed. In particular, the participants were recruited from August 2019 to February 2021 from curves fitness clubs located in areas across Taiwan. The ages of the participants at the time of the interviews were between 40 and 53 years old. Prior to data collection, ethical approval was obtained from the first author’s university. Moreover, all participants were asked to sign and submit consent forms and assured that their responses would remain confidential and used for research purposes only.

**Table 1:** Basic information of participants.

Code	Gender	Age	Current position
A	Female	53	General Manager of Curves
B	Female	40	Deputy General Manager of Curves
C	Female	40	Chief Marketing Manager of Curves
D	Male	47	Chief Brand Manager of Curves
E	Male	42	Chief Human Resource Manager of Curves
F	Female	45	Chief Finance of Curves

### Procedure and data collection

Semi-structured interviews were conducted to collect information from the participants. Each interview lasted about 40 to 60 min and was recorded on audiotape for analysis. The interviews were conducted with the aid of an interview guide (Patton, 2002), listing essential questions to ensure consistency. The interview questions were developed using the study’s overall broad research questions and were significantly influenced by prior research on female exercise consumption and motivations for exercise consumption (Patton, 2002; Conlin et al., 2014). Moreover, interview material was transcribed verbatim as soon as it was collected. Regarding the quality, consistency, and accuracy of data collection and analysis, the researchers were responsible for data collection, transcription, and interpretation. The moderator took a semi-structured approach by asking open-ended questions to elicit thoughts from participants (Patton, 2002). Sample questions included: ‘How do you conduct strategic planning and marketing mix development for the female fitness market?’, ‘What features do women like in physical training?’ and ‘What influences women to purchase a particular physical product regarding membership fees and strategic fitness finance plans?’ The interviewee signed an informed consent form prior to the interview. The confidentiality of the information was confirmed.

### Data analysis

This study used thematic analysis to analyze the descriptive data (Patton, 2002). Once coding was conducted, the first author generated a codebook containing a description of each node. The codebook was given to a second coder, who was a sports management doctoral familiar with constant comparison analysis and was informed of the purpose of this research. Transcripts were fractured sentence by sentence in the opening coding stage. During the coding process, we frequently discussed the code list and added codes that emerged through the process. The codes were then sorted to generate categories and sub-categories. The analysis focused on the relationships between categories and their subcategories (Lincoln and Guba, 1990; Patton, 2002). In addition, study participants were sent a copy of the transcript of their recorded interviews and were given a chance to review their comments, providing an opportunity to clarify statements and ensure they were portrayed accurately. In order to provide transparency, an outside researcher familiar with consumer behavior was asked to read the transcripts and code the documents independent of the primary researcher. Once this task was completed, the primary researcher met with this outside source and compared codes and themes. The final step was selective coding, in which the first author connected the categories, discovered their relationships, and developed a story based on marketing association theory and female marketing models (Petruzzello and Corbin, 1988; Patton, 2002). This method produced a more diverse and comprehensive picture of the findings. The credibility, transferability, dependability, and confirmability (Patton, 2002) were implemented by providing a summary of participants’ responses immediately after each interview by emailing interview transcripts and the analyzed data to participants and through systematic record-keeping and continuing evaluation of theories and findings. Moreover, based on Patton (2002), we adopted three reflexive methods to increase authenticity. First, the authors used field notes to record non-linguistic information regarding the interviewees’ actions, the interactions between the interviewer and the interviewees, and the interview conditions of each interview. Second, we increased the detail of the descriptions. In addition to the field notes, reflexive journaling was used to reduce the potential influence of the researcher’s subjectivities. Finally, during the data analysis, peer debriefing was conducted to verify the themes and sub-themes to ensure that consensus was reached regarding the results.

## Results and discussion

This study aims to examine the successful fitness training health model of a female fitness club. In addition, we contend that the existing social, mental, and physical aspects of servicescapes offered by gyms and fitness centers must be changed when marketing to women. Studies of the fitness marketing strategies of today’s fitness centers have shown the importance of different customer groups’ levels of satisfaction regarding the centers’ service quality. Most studies focused on customer satisfaction and service quality, but the four elements of the marketing mix and their role in the development of fitness centers’ marketing strategies have been often overlooked (Chang, 2020; Andreasson and Johansson, 2021; Wang and Chiu, 2022). This study describes marketing strategies for the female market by examining the combination of the four elements of the marketing mix. In this way, we show how the power of the innovation market is manifested through the marketing strategy applied to females.

### Product–product concept and management that put customers’ feelings first

Curves not only provides tangible products of sports courses; its main product is the intimacy the staff demonstrates when providing services, which includes caring about customers and establishing a sense of belonging. Curves competes with other traditional fitness centers with this level of intangible services.

*‘Curves provides services that include caring about its customers and establishing a sense of belonging. (D)*

*Services are one of the products of Curves. It emphasizes service and care. (F)*

*Curves is in the sports service industry. (E)’*

Curves has positioned itself in the sports service industry, its greatest product being the intimate degree of care it provides to its members. Trainers at Curves initiate caring for club members and create an atmosphere suitable for women to exercise, building emotional connections with female customers through dedicated interactions. They also make phone calls to further understand members’ personal needs, such as weight loss, body sculpting, and core conditioning, to provide products catering to each individual customer. These practices have helped Curves gain a foothold in the competitive fitness industry. Beetles and Harris (2005) indicated that when marketers are responsive enough to women’s sensitivities, the results can be surprising. This is because the type of product does not necessarily determine women’s purchasing intentions; the marketing approach does.

Curves has understood the personal needs of female customers and designed sports products dedicated to women, providing them with a pleasant experience with excellent services and a unique circuit-training-inspired gym setting. The interviews conducted in this study show that Curves advocates a 30-min workout with 12 pieces of gym equipment and 12 recovery boards. Members are encouraged to complete a cycle of the equipment and recovery in 24 min. Curves continues to add elements to existing fitness classes and design different classes to ensure that they are interesting, efficient, and convenient.

*‘Work out with 12 pieces of equipment and 12 recovery boards in 24 minutes to save time and ensure effectiveness! Extra cardiovascular games make products (i.e., classes) more interesting. (D)*

*Other products include advanced training for individuals, hydraulic gym equipment, and personal advanced training records. (C)*

*Pulses are measured every eight minutes to ensure members’ fat burning rates are at the optimal range of 60–70%. (B)’*

Curves’ product contents can be divided into the categories set forth by Sevastia and Nicholas (2014), core products, i.e., fitness classes for female customers; basic products, i.e., emphasizing fitness results; expected products, i.e., achieving fitness results by recording customers’ highest heart rates during their workouts; augmented product, i.e., customizing product contents by adding extra elements or designing exercise classes to make them interesting; and potential product, i.e., making female customers feel good through friendly interactions and building emotional connections. Every aspect of Curves is designed for women, from the size of each piece of hydraulic gym equipment to the atmosphere of the workout environment.

*‘The equipment is designed for female body sizes; the workout atmosphere and the workout circuit design are all customized for women! The entire system is developed based on female needs. (A)*

*The equipment, workout atmosphere, and environment are designed by the founder according to the needs of a 45-year-old woman. (D)*

*Chat with members for 15 minutes and build up relationships with them when taking body measurements. (F)’*

This study found that Curves has applied precise segmentation to its marketing targets in the fitness market. It has targeted a certain consumer group and designed customized products for them. For example, it offers a 15-min body measurement session for members every month and helps them set fitness targets. In addition to fitness classes, Curves develops functional peripheral products along with monthly promotions like giveaways or awards for members who meet certain targets. Curves has managed to create its product mix successfully. Curves has applied the product element of the marketing mix by taking the initiative to care for its members, understanding members’ needs, and assisting them in achieving goals to build a sense of belonging. This echoes the ideas of past studies that marketing to women should take female needs and features into consideration and that market segmentation should be applied to women. The marketing approach determines women’s involvement rather than the types of activities offered. Marketers who are responsive enough to understand women’s sensitivities manage to attract female customers (Cuneen and Claussen, 1999; Samaha et al., 2014).

### Price-transparent pricing guidelines that gain customers’ trust

According to the respondents, Curves charges a fixed fee and adheres to a transparent pricing structure to build mutual trust with its customers and to help them maintain general positive feelings about their purchases. The fee is an appropriate mean value calculated from the price index and national wage rates of Taiwan. On the other hand, prices of peripheral products are assessed according to the production costs, brand fees, freight rates, and prices of substitutes in the market.

*‘Emphasis on high transparency of prices (A)*

*Use the price index and wage rates of Taiwan as benchmarks (D)*

*Promotion activities: attaining more than 50% profit with three products (B)’*

Curves focuses on helping women become a better version of themselves through sports. Roussel et al. (2003) concluded from studying female consumer behavior that there are four factors necessary for resonating with women: customization for modern women; emotions that draw women’s attention to your brand; a corporate image of helping customers shine; and transparency. Transparency, rather than femininity, is the best approach for marketing to women.

Conlin et al. (2014) suggested that pricing strategies should be considered after evaluating the cost of a product. Enterprises need proper pricing tactics to generate the greatest investment returns and to control their operations’ efficiency. Consistent with the findings of past studies, Curves’ pricing strategies have enabled stable profit increases (Wang and Chiu, 2022). Respondents stated that the profit formula in Curves’ operating model consists of word-of-mouth marketing (i.e., word-of-mouth advertising among customers), which accounts for up to 50% of monthly profit; brand marketing (i.e., using signboards, the internet, and advertisements to increase brand impressions), which accounts for up to 25% of monthly profit; and proactive client development, including distributing flyers and newsletters and direct mail, which accounts for up to 25% of monthly profit. This echoes the findings of Teik (2015) on fitness clubs’ business, which stated that fitness clubs mainly promote themselves through newsletters, magazines, and mailed flyers. Curves’ marketing strategies also include providing free giveaways, free trial offers, and price discounts to create new markets. The interview conducted for this study revealed two key elements of Curves’ marketing approach: developing people’s interest in sports and then providing them with services of good value. Similarly, Curves has attracted female customers by providing professional coaching at the start of their membership and gained their support through a fixed-price monthly membership along with marketing activities afterward.

### Place-establishing standard operating procedures by stringently evaluating the surrounding environment of the stores

Marketers must choose a suitable delivery system so that consumers can easily get the products they need through the system. They must consider factors such as business environmental assessment, the enterprise itself, product positioning, and sales channels (Hans and David, 1999; Samaha et al., 2014 Haenlein and Libai, 2017). In terms of its strategy in selecting a location for its gym, Curves focuses on operating in a community and building its own marketing research process on distribution channels to guarantee the operating profit of its chains.

*‘Firstly, the location has to be bustling. Secondly, there should be an adequate female population of 80,000 for the chain store to operate in the community. (D)*

*Counting the number of women aged 30-55 passing by the nearby large, middle, and small chain stores and those stores females regularly visit. (G)*

*Each store uses two report forms to run cross-validation on management quality. (A)’*

Curves’ strategy is consistent with the views of Samaha et al. (2014) that some degree of benign channel conflicts is constructive and that effective management of channel conflicts has become increasingly important. Curves has segmented the market at the same distance to protect each franchisee’s right to make profits and to minimize damages from channel conflicts. Curves has made further efforts to design its placement strategy evaluation forms to have a precise understanding of the commercial zones nearby and the population in the vicinity of each store. It has also required each store to make appointments with 1,500 customers for trials before the store opens so as to meet Curves’ target to break even in the first week of operations. Moreover, Curves headquarters gathers statistics on monthly sales of peripheral products in each store to reduce inventory costs. The headquarters and franchisees join forces to plan monthly promotion activities like giveaways or special sales for members to save ordering costs and time and to increase profit.

*‘All members in Taiwan can exercise in different stores in the chain (F)*

*Cloud computing nowadays is very convenient. The system is able to notify members of the locations of nearby stores and offer them free trials. (E)*

*Products become our marketing tools. (B)*

*The headquarters will stock 10% of the products to reduce inventory by a substantial amount. (C)’*

Curves’ placement strategy emphasizes product consistency through stringent internal management of the physical arrangement of its gyms. Curves’ management also controls the provision of specific sales channels for peripheral products, so the stores and headquarters can operate in a mutually beneficial way to make profits. Curves has managed to reduce the cost burden and improve the profit performance of the enterprise with its unique place marketing model.

### Promotion-being aware of female customers’ lives to formulate comprehensive promotion plans

Promotion is the process of creating an appealing situation to arouse consumers’ buying motives in order to sell products. Markula (1995) pointed out that among the various marketing strategies, there is a wide spectrum of promotion methods presented in different forms in the marketing model. Marketing tools that directly stimulate spending are used to package the products in comprehensive promotion plans, promptly making the products more valuable and desirable in the consumers’ eyes. Curves’ promotion strategies include distributing flyers, using direct mail, issuing newsletters, and operating an online fan page to take the initiative in contacting customers with the aims of enhancing brand image, increasing brand awareness, and developing a market of target customers in a proactive manner.

*‘Aggressively develop customers by distributing flyers and newsletters, signing up on the streets, online advertising, and bus advertising to increase brand awareness (D)*

*Proactively talk to women and invite them in for a free trial (G)*

*Keep an eye on female websites and the official fan page on Facebook (A)*

*Utilize members’ referrals, word-of-mouth marketing, and other referrals for the sharing effect (C)’*

The reason that the female consumer market today is stronger than that of males is that once a woman is content with a product or service, she will recommend it to others by word of mouth, multiplying the number of customers as a result; this is more powerful than individual purchases (Hardin et al., 2008; Mills and Williams, 2016). Curves understands the behavior of female customers and has analyzed the factors that determine women’s spending decisions. Understanding the features of female customers is the key to dominating the female fitness market. Curves has also emphasized operating in the community and focusing on word-of-mouth advertising among members to consolidate the brand’s influence in boosting women’s physical health, beauty, and self-confidence. This echoes the view of Hans and David (1999) that sports centers should respond to customers’ demands through promotion activities in order to encourage interactions among customers, thus enhancing the word-of-mouth advertising effect among members.

*‘The logo of Curves is printed on the peripheral products to enhance the brand image and to maintain brand consistency. (D)*

*Participating in charity events is favorable to the brand image. The brand image will be more and more positive. (F)*

*Operating in the community highlights the paramount importance of word-of-mouth advertising. (E)’*

Curves has achieved its phased promotion targets one by one with its promotion tactics. It has successfully penetrated the female fitness market by applying the concept of interlinked promotion to different phases of promotion activities. The booming sports industry is developing in a rapid and diverse way. Female customers have become a powerful consumer group in the fitness market (Andreasson and Johansson, 2021). The slogan ‘Amaze Yourself’ is printed on the direct mail of Curves to convey to female consumers that regular exercise can lead to stunning improvements. This further emphasizes how Curves satisfies women’s demands for exercise and how Curves is able to help women lead better lives. The research findings show that Curves has integrated its promotion stages in a progressive manner with various marketing communication tools like mass media, advertisements, and word-of-mouth advertising among clients. Curves has analyzed women’s workout needs and features and formulated diverse and effectively integrated promotion plans that share the same values with the female consumer group, arousing their emotional resonance.

### Derive the key success factors for curves to continue operating in Taiwan

Private fitness clubs are quite a competitive industry in Taiwan. With a population of 23 million, the market is actually quite saturated. From the very beginning, Curves targeted females, saying that they would directly give up the other half of Taiwan’s market, which would be more restrictive. In order to stand out in the competitive fitness industry, Curves has to compete not only with general fitness clubs but also with other leisure and recreational industries that women love, such as yoga, aerial yoga, squash, and aerobic dance, among others. Therefore, Curves must be unique in its stable and long-term growth in Taiwan. Below is a summary of the key success factors that distinguish Curves from other fitness clubs of a similar nature.

### Become a second home for consumers

Curves makes women feel as comfortable at home but also allows women to get rid of the complexity of housework. Sometimes, the purpose of women engaging in leisure activities is to temporarily get rid of cumbersome housework, children, or husbands. It allows them to be able to have a space of their own and take a breath.

*‘Curves is a home away from home for women to be better selves and amaze themselves by playing sports. (D)*

*‘Curves put members’ feelings first. It proactively phones members up to make them feel important and arouse their desire and motivation to work out. (A)’*

*It is their pleasure to transform their members’ lives by achieving the corporate mission.*

*‘Emphasis on being a family with members (E)*

*Building a sense of belonging is the core of the marketing model. (A)’*

The key to creating a marketing model that wins over female customers lies in a deep understanding of the factors that affect their demands. Female consumers are more emotional, so the marketing approach should include having good interactions and building emotional connections with them to arouse their resonance and further increase their willingness to buy (Hardin et al., 2008; Seidman, 2013; Ratten, 2016). Curves’ marketing to women focuses on the characteristics and demands of female members so as to design marketing activities in different seasons and months to attract their attention and participation. Its approach of encouragement, care, companionship, constant and positive communication, and word-of-mouth advertising encourages its members to build good exercise habits (Paul et al., 2004; South and Lewis, 2011). This indirectly makes Curves a part of their life and generates benefits for the female fitness market in Taiwan.

### Connect with female-related charities

Taiwanese are often quite devoted to charity and public welfare undertakings. Women, in particular, are more likely to participate in charitable activities with empathy. Curves has created a favorable corporate image through charity and community engagement. Consumers’ rights are guaranteed by its transparent pricing guidelines.

Curves has targeted its marketing at certain consumer groups and organized monthly promotion activities, such as anti-breast cancer charity events, and cooperated horizontally with Common Health Magazine to formulate price promotions.

*‘Collaborate with Taiwan Breast Cancer Foundation, offering discounts on breast cancer screening, pap smear tests, and membership fees (G)*

*Monthly promotion activities as a reward system for members (B)’*

This study found that when Curves applies its pricing strategy, it targets related charity events for potential customers and designs different price promotion activities every month. For example, it collaborated with the World Breast Cancer Foundation to host a Pink October event, encouraging members to carry out breast cancer screening and Pap smear tests, and integrated its membership fee promotion policies with this collaboration. In addition to mobilizing its members to raise funds for charities, Curves has encouraged its members to assist in the organization of such events. Members become more emotionally invested in the cause of the charity, and Curves is able to reap the benefits of the positive image it has built by associating itself with the charity.

### Design a marketing theme event that touches women

Curves has applied diverse promotion methods. It proactively develops a list of new clients, takes the initiative to introduce the courses to female passers-by, provides free trial offer vouchers to attract female consumers, and uses relatively aged women as advertising characters to capture the attention of the target consumer group. Curves also distributes newsletters to increase its brand awareness, advertises on specifically female websites, and operates a fan page on Facebook to maximize the spread of brand influence. Shaw (2012) suggested that in order to achieve a promotion target, an enterprise must formulate extensive promotion plans and develop a comprehensive marketing mix. To this end, Curves draws up various promotion ideas every month to arrange and design a range of activities and games for its members. In addition to extensive sports courses, it focuses on the implementation of an award mechanism for members by giving away peripheral products through games and activities.

*‘Design distinctive activities and games so that members will think it effective, fun, and not lonely (D)*

*The promotion plans take into account the issues that draw women’s attention the most (A)*

*January and February: point collection for an automobile; March: anniversary gift pack; April: food donation activity in collaboration with the Garden of Hope Foundation; May and June: Mother’s Day giveaway; July and August: 500,000-time workout challenge; September: boot camp; October: worldwide anti-breast cancer Pink October; November and December: grassland adventure point collection (C)’*

**Figure 1:**
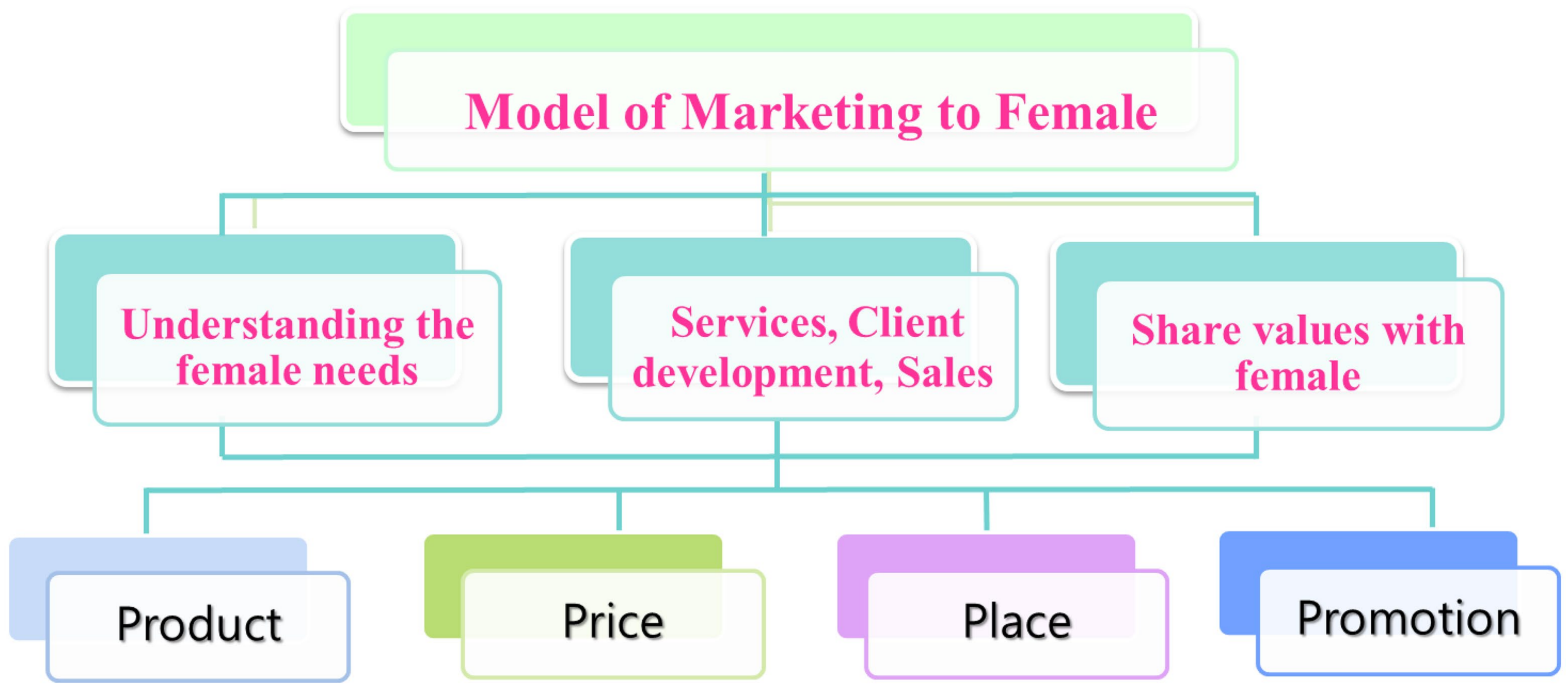
Female’s innovation marketing mix.

Curves’ business model for formulating marketing strategies specifically for female customers is summarized in Figure 1.

## Conclusion

This study explored the female marketing mix model of the female-only gym business. We conducted a series of semi-structured interviews to investigate three questions related to the female fitness marketing strategy. Through the first research question, we analyzed Curves’ internal operation model for female fitness marketing. We observed functional service, developing clients, generating scale, and reporting to management. Using the second research question, we examined how the characteristics of the marketing mix strategy were attractive to the female fitness group with product, price, place, and promotion. With the third research question, we examined Curves’ model of marketing to women. The relationships we observed are outlined in Figure 1, which indicates the marketing mix structure of the female fitness group. Our paper provided discussions of the structure of fitness marketing strategies for women.

This research adopted a third-party viewpoint to explain the marketing strategy and specific practical marketing strategies for women implemented by the Curves female-only gym in Taiwan. The research used purposive sampling to select seven executives in the headquarters of the Curves female-only club and conducted interviews to discuss the business’ marketing mix. The results showed that (1) Curves created an atmosphere that made the gym feel like a second family and fostered a sense of belonging for customers by focusing on female members’ experiences and customizing their product concept, exercise programs, proactive care, and services; (2) Curves set consistent and transparent pricing to obtain customer’s trust and create a sense of identity; (3) Curves established SOP and placed emphasis on its brand consistency management for its chain stores, and (4) Curves had a seasonal promotion and peripheral products which aimed to attract female sentiments and attentions.

Overall, this study highlights the importance of fitness organizations setting their marketing strategies to focus on females to increase profits. Moreover, future studies are suggested to follow the results of this study and use different methods or multiple viewpoints to discuss more marketing strategies for one gender or various customer groups. Our study highlights that in multi-cultural settings, a new contextual perspective for the female marketing model, as the success of the fitness marketing mix model has important implications for the female consumption market. Curves is gaining a foothold in the fitness market in Taiwan, helping women develop a new and healthy lifestyle along the way.

### Theoretical implications

Our research emphasizes that service providers should carefully examine the expectations of female clients for exercise in a multicultural context. We found that the fundamental principle in marketing to female customers is to put the customer’s feelings first. Female customers’ trust can be gained by providing high-quality services and products with transparent pricing and charging guidelines. Curves implements standard operating procedures with stringent evaluations of the surrounding environment of its stores.

Furthermore, Curves is aware of the concerns that appeal to women and has established comprehensive promotion strategies by creating various seasonal events. This is the key to dominating the female fitness market. Thus, the model of marketing to women has utilized the trends of the fitness market (Shaw and Amis, 2001; Wang and Chiu, 2022). With an acute sense of the changes in women’s demands for fitness clubs, Curves has created a marketing mix with specified contents and an internal operating approach customized for women. It has created a blue ocean in the fitness industry and set a benchmark for the female fitness market.

Finally, the current findings support a new contextual perspective for the female marketing model. It is necessary to pay extra attention to the reciprocal values of the drafting marketing mix strategy, as the success of the fitness marketing mix model has important implications for the female consumption market.

### Practical implications

Curves has managed to identify the constant changes in the female fitness market and employ diverse marketing strategies to cater to the ever-changing demands of the female market. This has enabled Curves to survive and gain a foothold in the fitness market in Taiwan, helping women develop a new and healthy lifestyle along the way. This research suggests that Curves, which emphasizes the importance of service quality, should continue enhancing the human resources of its franchisees by providing them incentives and rewards. Fitness trainers, who play a vital role in the enterprise, should be respected and provided with long-term assistance in planning their personal careers. Their career development can thus be safeguarded, and the turnover rate of trainers in chain stores can be reduced. The trainers will also be able to maintain their passion for providing fitness instruction, and the high service quality can then be maintained. The core values and business philosophy of Curves will be implemented in a positive and proactive manner.

In addition, fitness chain operators should be set up to strengthen communication between franchisees and members so that the head office can communicate directly with members to understand their needs. Moreover, to understand the needs of the members so that the overall marketing approach can be adjusted to meet the needs of the customers and enhance their repurchase intention (Wang and Chiu, 2022). The head office should be able to communicate directly with its members to understand their needs so that it can adjust its overall marketing approach to meet customer needs and increase their willingness to spend.

Conversely, the head office should strengthen its role as a third-party supervisor and coordinator in cooperating with franchise owners, for example, through a franchisee association platform. The Association of Franchise Owners platform. In the event of a conflict between shops and customers, or when there is a difference in business philosophy between the head office and the franchisee, third-party monitoring and coordination are essential. Thus, the role of the third party should be to protect the rights and interests of the head office, franchise owners, members, and co-ordinate. This enables fitness operators to establish a systematic operating organization and enhance the effectiveness of the group’s operations.

### Limitations and future research

We urge caution when interpreting the findings, as the participants were chosen purposively and are the ones that were able to answer the research questions. Replicating the study with participants who have different characteristics may not yield the same findings. For example, a different sports industry or a group of consumers may have a different set of marketing model associations. In addition, these findings are in cultural terms, and they may change if other methods are used. Semi-structured participants tended to censor their particular comments and fit into the majority view (Conlin et al., 2014).

It is likely that our results have not fully revealed the extent of attributes because of in-depth individual interview effects. In our study, we held individual interview discussions at places where participants were aware. Through these efforts, we hope to provide a safe setting for open sharing and provide participants sufficient time to open up to one another. Future studies should investigate whether the more conservative Eastern culture, namely Chinese culture, has an effect on the success of the marketing strategy of Curves. Eastern culture has been known to be more conservative and patriarchal, and while it does not necessarily belittle the strength of women. It does not place a heavy emphasis on women being self-determined and self-aware. As such, women are constantly under the judgmental eyes of a patriarchal society, and gyms like Curves may provide them with a temporary sanctuary where they can be amongst their peers without the constant gaze of men.

In this sense, Curves would be appealing to women in the more conservative Eastern culture, but whether the same appeal exists in the more liberal and egalitarian Western culture remains to be seen, and future studies exploring this difference in cultures may show interesting findings.

## Data availability statement

The original contributions presented in the study are included in the article/supplementary material, further inquiries can be directed to the corresponding author.

## Author contributions

F-JW, C-HH, and T-TH: conceptualization, formal analysis, investigation, and funding acquisition. F-JW: methodology, writing—review and editing, data curation, project administration, and writing—original draft preparation. All authors contributed to the article and approved the submitted version.

## Funding

This work was supported by the National Science and Technology Council, Taiwan (award number: NSTC 111-2410-H-032-030-MY2).

## Conflict of interest

The authors declare that the research was conducted in the absence of any commercial or financial relationships. The authors have no conflicts of interests to declare.

## Publisher’s note

All claims expressed in this article are solely those of the authors and do not necessarily represent those of their affiliated organizations, or those of the publisher, the editors and the reviewers. Any product that may be evaluated in this article, or claim that may be made by its manufacturer, is not guaranteed or endorsed by the publisher.
